# The Protein Maker: an automated system for high-throughput parallel purification

**DOI:** 10.1107/S1744309111028776

**Published:** 2011-08-13

**Authors:** Eric R. Smith, Darren W. Begley, Vanessa Anderson, Amy C. Raymond, Taryn E. Haffner, John I. Robinson, Thomas E. Edwards, Natalie Duncan, Cory J. Gerdts, Mark B. Mixon, Peter Nollert, Bart L. Staker, Lance J. Stewart

**Affiliations:** a Seattle Structural Genomics Center for Infectious Disease (http://www.ssgcid.org), USA; b Emerald BioStructures, 7869 NE Day Road West, Bainbridge Island, WA 98110, USA; c Emerald BioSystems, 7869 NE Day Road West, Bainbridge Island, WA 98110, USA

**Keywords:** automation, chromatography, high-throughput protein purification, influenza virus, Protein Maker, SSGCID, structural genomics

## Abstract

The Protein Maker is an automated purification system developed by Emerald BioSystems for high-throughput parallel purification of proteins and antibodies. This instrument allows multiple load, wash and elution buffers to be used in parallel along independent lines for up to 24 individual samples. To demonstrate its utility, its use in the purification of five recombinant PB2 C-terminal domains from various subtypes of the influenza A virus is described. Three of these constructs crystallized and one diffracted X-rays to sufficient resolution for structure determination and deposition in the Protein Data Bank. Methods for screening lysis buffers for a cytochrome P450 from a pathogenic fungus prior to upscaling expression and purification are also described. The Protein Maker has become a valuable asset within the Seattle Structural Genomics Center for Infectious Disease (SSGCID) and hence is a potentially valuable tool for a variety of high-throughput protein-purification applications.

## Abstract 2

The Protein Maker instrument addresses a critical bottleneck in structural genomics by allowing automated purification and buffer testing of multiple protein targets in parallel with a single instrument. Here, the use of this instrument to (i) purify multiple influenza-virus proteins in parallel for crystallization trials and (ii) identify optimal lysis-buffer conditions prior to large-scale protein purification is described.

## 1. Introduction

**Figure 1:**
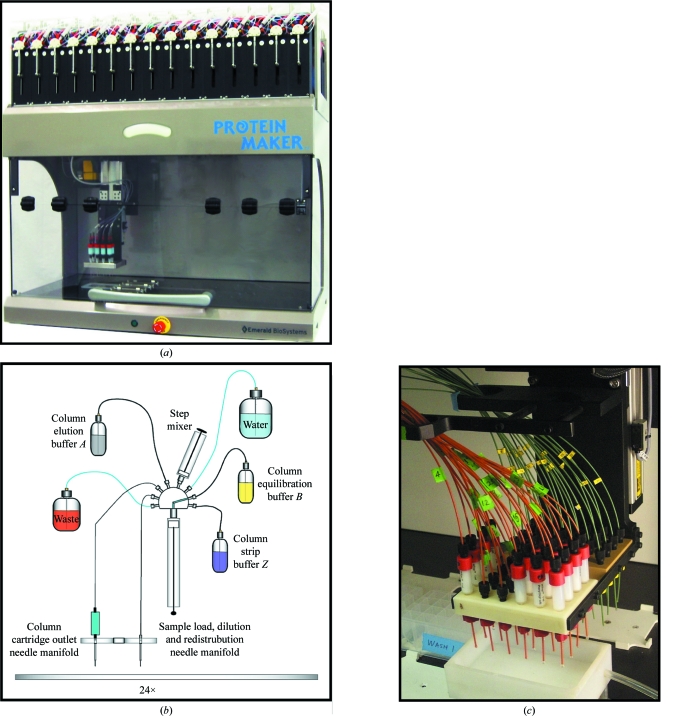
The Protein Maker instrument with syringe valves, liquid-handling sample manifold and deep-well plate deck (*a*). In the depicted configuration, 15 of the 24 syringe valves are at the front, with the remaining nine at the back (not shown). Also depicted is a schematic drawing of the plumbing for each individual nine-port valve (*b*) and a close-up image of the sample-load and primary purification manifolds with 24 × 1.0 ml purification columns in place (*c*). All 24 valves are individually lined and independently operated, thus allowing up to 24 sample-uptake lines and purification columns in a single run.

The end goal of structural genomics is the rapid generation of three-dimensional structures obtained from atomic level resolution studies of pure proteins (Xiao *et al.*, 2010 ▶; Elsliger *et al.*, 2010 ▶; Watson *et al.*, 2007 ▶; Bonanno *et al.*, 2005 ▶). Therefore, purifying protein targets in the shortest amount of time maximizes the number of potential candidates for further processing. Gravity-based, high-pressure and ‘fast protein’ liquid-chromatography (HPLC and FPLC) methods have been implemented on various scales to purify individual targets. Although suitable for a research environment (Walls *et al.*, 2011 ▶), most standard instrumentation does not allow parallel purification or testing to investigate a large number of purification conditions simultaneously. To address this critical need on a structural genomics scale, much research and development has gone into the creation of pipelines designed to deliver the necessary high-quality materials at a rapid pace (Kim *et al.*, 2008 ▶; Cymborowski *et al.*, 2010 ▶; Stols *et al.*, 2002 ▶; Dieckman *et al.*, 2002 ▶; Steen *et al.*, 2006 ▶). To facilitate rapid purification of proteins for structural genomics, we have developed the Protein Maker, a high-throughput parallel liquid-chromatography system that is capable of purifying up to 24 protein targets in a single unattended run (Fig. 1 ▶). The result is the purification of multiple targets with identical or individualized buffer systems, decreasing the time required for purification while potentially increasing protein yields through time-efficient buffer optimization.

The Seattle Structural Genomics Center for Infectious Disease (SSGCID) has a mandate to generate approximately 100 protein structures each year from a variety of Category A, B and C pathogenic organisms, as listed by the National Institute of Allergy and Infectious Diseases (NIAID; Van Voorhis *et al.*, 2009 ▶; Myler *et al.*, 2009 ▶). The targets selected for study are proven or putative points of therapeutic intervention and include a high-value subset of proteins requested by external scientists from the infectious disease research community. Multiple sequence variants are queued for most targets to increase the likelihood that at least one gene product will express, purify and become suitable for study by NMR spectroscopy or X-ray crystallography. In this communication, we introduce the Protein Maker and describe its use in purifying five C-terminal domain subunits of the polymerase basic protein 2 (PB2) from two different subtypes of influenza virus. Specific C-terminal domain mutations in the PB2 component of the viral polymerase heterotrimer have been associated with different levels of human virulence, giving structural characterization of this protein a potentially important role in the prevention of future influenza pandemics (Guilligay *et al.*, 2008 ▶; Subbarao *et al.*, 1993 ▶; Tarendeau *et al.*, 2008 ▶; Yamada *et al.*, 2010 ▶). All five C-terminal domain PB2 (CPB2) variants were purified in a single run of the Protein Maker and three of them crystallized, yielding one crystal system with sufficient quality for structure determination by X-­ray diffraction and subsequent deposition in the Protein Data Bank (PDB; Berman *et al.*, 2000 ▶, 2003 ▶). Another application of the Protein Maker is in scouting for optimal cell-lysis buffer conditions, an earlier step in the pipeline used to maximize protein yields during scale-up procedures. This is of particular value for proteins which exhibit high levels of expression but appear to be insoluble under standard lysis-buffer conditions. In this report, we also detail scouting experiments in which a single batch of cells was split into 12 pools and lysed by sonication in 12 different buffer conditions, followed by parallel purification with the Protein Maker. The protein in this case was cytochrome P450 51 A1 (CYP51A1) from *Coccidioides immitis*, the organism which causes coccidioidomycosis or San Juan Valley fever (Crum *et al.*, 2004 ▶; Hector & Laniado-Laborin, 2005 ▶; Galgiani, 1999 ▶). Both studies highlight the advantages in using a parallel purification platform such as the Protein Maker in a high-throughput structural genomics pipeline.

## 2. Experimental methods

### 2.1. Expression and purification of PB2 viral polymerase subunits

Five constructs were designed spanning the C-terminal domain of influenza virus A polymerase basic protein 2 (CPB2), one component of the heterotrimeric viral polymerase. These CPB2 constructs were derived from two genetic sources: an H3N2 swine-flu subtype isolated in Japan and an H7N7 equine subtype originating in the Czech Republic (see Table 1 ▶). Each of the five constructs was cloned into a modified pET28 vector system engineered to donate an amino-terminal 6×His-Smt fusion tag (Mossessova & Lima, 2000 ▶) and a site-­specific protease cleavage site to the open reading frame using Polymerase Incomplete Primer Extension (PIPE) cloning (Lorimer *et al.*, 2009 ▶). The N-terminal sequence of all five gene products was MSHHHHHHSGEVKPEVKPETHINLKVSDGSSEIFFKIKKTTPLRRLMEAFAKRQGKEMDSLRFLYDGIRIQADQTPEDLDME­DNDIIEAHREQIGGS followed by the first amino acid of the desired influenza A viral protein construct. Each clone was expressed in *Escherichia coli* BL21 (DE3) cells grown in autoinduction medium (Terrific Broth plus Novagen Overnight Express System 1) using a LEX bioreactor at 293 K for approximately 65 h. Cells were collected by centrifugation at a relative centrifugal force (RCF) of 32 960*g*. The supernatant was decanted and the cell paste was flash-frozen in liquid nitrogen prior to storage at 193 K. The cell paste for each CPB2 protein was then thawed and resuspended in lysis buffer at a 1:5 mass:volume ratio. The lysis buffer (500 ml) consisted of 25 m*M* Tris pH 8.0, 200 m*M* NaCl, 50 m*M* arginine, 10 m*M* imidazole, 0.02% CHAPS, 0.5% glycerol, 1 m*M* Tris(2-carboxyethyl)phosphine (TCEP), 100 mg lysozyme, 500 U Benzonase and one Complete Protease Inhibitor Cocktail tablet (Roche). The cells were resuspended by vigorous stirring for 30 min at 277 K and mechanically lysed on ice using a Misonix sonicator (70% power, 2 s on/1 s off pulses, 3 min total). The crude lysate was clarified immediately after sonication by centrifugation at 18 000*g* RCF for 35 min at 277 K. The clarified supernatant was decanted and stored at 277 K prior to purification with the Protein Maker.

All clarified lysate samples were brought to 105.0 ml by addition of buffer *A* (25 m*M* Tris pH 8.0, 200 m*M* NaCl, 50 m*M* arginine, 10 m*M* imidazole, 0.25% glycerol and 1 m*M* TCEP). One 5.0 ml HisTrap FF nickel-chelate column (GE Healthcare) for each CPB2 sample was attached to a separate line on the gantry of the Protein Maker, with the following steps conducted in parallel for all samples. Each purification step was conducted in 5.0 ml volumetric increments, utilizing a flow rate of 1.5 ml min^−1^. For the first run of the Protein Maker (‘Nickel 1’), each column was first washed with 20.0 ml Milli-Q water to remove the ethanol-based storage buffer. Each column was then washed with 5.0 ml buffer *B* (25 m*M* Tris pH 8.0, 200 m*M* NaCl, 500 m*M* imidazole and 1 m*M* TCEP) followed by equilibration with 25.0 ml buffer *A*. Resuspended cell lysates were then loaded onto individual columns followed by a 15.0 ml wash with buffer *A*. Each protein sample was then eluted in three steps: 5.0 ml at 30 m*M* imidazole, 5.0 ml at 206 m*M* imidazole and 10.0 ml at 500 m*M* imidazole, using 96:4, 60:40 and 0:100 ratios of buffer *A*:buffer *B*, respectively. These buffers were drawn from separate individual stocks and actively mixed in the syringe valve prior to column loading (Fig. 1 ▶). Elution fractions were collected separately in deep wells of a Whatman 24-well plate positioned on the deck of the Protein Maker. After the final elution step, all columns were re-equilibrated with 100.0 ml buffer *A* as a temporary storage condition. Elution fractions were tested for the presence of protein using 4–12% Bis-Tris SDS–PAGE gels (Invitrogen). Fractions containing the protein of interest with a minimum of impurities were pooled and stored at 277 K.

**Figure 2:**
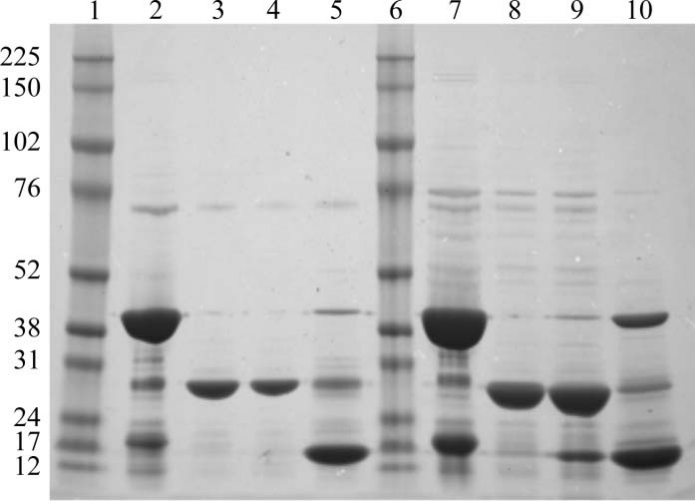
SDS–PAGE results for InvaB.07055.c (lanes 2–5) and InvaC.07055.b (lanes 7–­10) during nickel-chelate chromatography purification on the Protein Maker. Lanes 1 and 6, molecular-weight markers (labeled on the left in kDa); lanes 2 and 7, pooled protein from Nickel 1; lanes 3 and 8, flowthrough of cleaved protein in buffer *A* from Nickel 2; lanes 4 and 9, buffer *A* wash from Nickel 2; lanes 5 and 10, removal of 6×His-Smt tag, 6×His-tagged protease and uncleaved protein with buffer *B* from Nickel 2.

Each of the five eluted and pooled CBP2 protein samples was brought to 10.0 ml in buffer *A* and treated with 50 µl 1.0 mg ml^−1^ 6×His-tagged ubiquitin-like protease 1 (Ulp1) overnight to cleave the N-­terminal 6×His-Smt fusion tag. Ulp1 cleaves the protein between the first residue of each viral target and the C-terminal serine of the QIGGS tag sequence, leaving no remnant of the tag on the protein. Each sample was then dialyzed against 2.0 l buffer *A* for a minimum of 4 h to reduce the concentration of imidazole prior to further purification on the Protein Maker. The second run on the Protein Maker (‘Nickel 2’) also employed 5.0 ml volumetric increments, but at a reduced flow rate of 1.0 ml min^−1^ for all ensuing steps. Samples were loaded onto the Protein Maker and passed through the same HisTrap FF columns as in the first run. During Nickel 2, the cleaved 6×His-Smt tag, the 6×His-tagged protease and any un­cleaved target proteins bound to the column, while fully cleaved CPB2 eluted in the flowthrough. The columns were then washed with 3.0 ml buffer *A* followed by 5.0 ml buffer *B* to remove all 6×His-tagged and nonspecifically bound proteins from the column. Flowthrough, wash and elution fractions from Nickel 2 were collected separately and analysed by SDS–PAGE for comparison with the initial pooled protein from Nickel 1 (Fig. 2 ▶). All five influenza CBP2 proteins were successfully cleaved in this process and were pooled and concentrated to a volume of 5.0 ml for subsequent size-exclusion chromatography.

A Sephacryl S-100 10/300 GL column (GE Healthcare) was prepared by equilibration with 200 ml SEC buffer (25 m*M* Tris pH 8.0, 200 m*M* NaCl, 1.0% glycerol, 1 m*M* TCEP) at 0.5 ml min^−1^ using an ÄKTApurifier system (GE Healthcare). A set of 5 × 10.0 ml superloops was used to stagger each sample injection over the SEC column in one continuous run. A UV-absorbance trace at 280 nm was used to analyze column eluents and determine the presence of protein as well as potential contaminants. Samples were collected in 3.0 ml fractions, analysed by SDS–PAGE, pooled and concentrated to approximately 20 mg ml^−1^ using Amicon Ultra 10 kDa molecular-weight cutoff centrifugation tubes. After reaching the target con­centration, each sample was divided into 100 µl aliquots, flash-frozen in liquid nitrogen and stored at 193 K prior to crystallization experiments.

### 2.2. Expression and purification of cytochrome P450 51 A1 from *C. immitis*

Cytochrome P450 51 A1 from *C. immitis* (CYP51A1) spanning residues 36–490 (CoimA.07054.l) was cloned into a modified pET28 vector with an N-terminal 6×His-Smt tag and cleavage site using PIPE cloning as described above. CYP51A1 was then expressed and the cells harvested and stored in the same manner as the CPB2 proteins described in the previous section. The cell paste for a single batch of CYP51A1 was divided into 12 aliquots of 3.0 g each for lysis-buffer testing. Each portion was first resuspended in 30.0 ml of a different lysis buffer on ice (Table 2 ▶). Each aliquot was then stirred for 30 min at 277 K followed by mechanical lysis on ice with a Branson sonicator (70% power, 2 s on/1 s off pulses, 3 min total). Crude lysates were spun at 32 960*g* for 35 min at 277 K to remove cell debris. The clarified lysates were decanted and brought to 35.0 ml total volume, followed by incubation at 277 K for 15 min, prior to purification with the Protein Maker.

**Figure 3:**
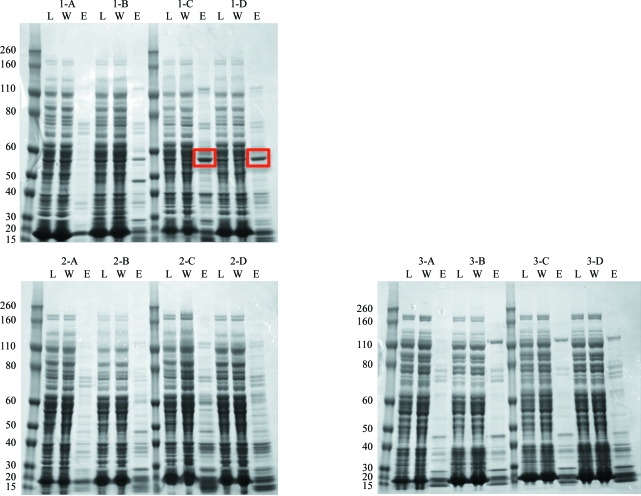
SDS–PAGE analysis of 12 different cell-lysis buffer conditions described in Table 2 ▶ for CYP51A1. Lanes correspond to the load (L), wash (W) and elution (E) cycles conducted in parallel on the Protein Maker, with the same molecular-weight standards throughout (labeled on the left in kDa). Cell-lysis buffer scouting resulted in two conditions with optimal yields after affinity chromatography (red boxed bands).

The following purification steps employ the same load, wash and elution buffers for all 12 samples to allow direct comparison of protein yields across different cell-lysis buffer components. 12 1.0 ml HisTrap FF (GE) columns were attached to the Protein Maker gantry, rinsed with 20.0 ml Milli-Q water, washed with 5.0 ml buffer *D* [50 m*M* 4-(2-hydroxyethyl)-1-piperazineethanesulfonic acid (HEPES) pH 7.5, 200 m*M* NaCl, 500 m*M* imidazole] and equilibrated with 25.0 ml buffer *C* (50 m*M* HEPES pH 7.5, 200 m*M* NaCl, 10 m*M* imidazole, 50 m*M* arginine, 1 m*M* TCEP, 0.25% glycerol) at 2.0 ml min^−1^. All clarified lysates of CYP51A1 were then loaded, followed by a wash with 30.0 ml buffer *A* at 2.0 ml min^−1^. Each sample was eluted in three steps at 0.75 ml min^−1^: 5.0 ml at 30 m*M* imidazole, 5.0 ml at 206 m*M* imidazole and 10.0 ml at 500 m*M* imidazole, using 96:4, 60:40 and 0:100 ratios of buffer *C*:buffer *D*, respectively. Elution fractions were collected separately in deep wells of a Whatman 24-well plate positioned on the deck of the Protein Maker (Fig. 1 ▶) and analysed by SDS–PAGE for optimal lysis-buffer components (Fig. 3 ▶).

## 3. Results and discussion

### 3.1. High-throughput parallel purification with the Protein Maker

The Protein Maker (US Patent No. 6818060, Emerald Bio­Systems) is a 24-channel parallel liquid-chromatography system developed specifically for high-throughput protein purification and related structural genomics pipeline applications. The instrument has 24 precision intake nozzles and syringe pumps, each with a nine-port valve allowing up to six different intake solutions per line (Fig. 1 ▶). Each port has a 5.0 ml syringe barrel to allow individualized step-gradient buffer mixing, with a maximum flow rate of 20 ml min^−1^ but typically run at 0.5 to 2.0 ml min^−1^ with the same flow rate in each line. Each channel has a column and resin capacity of 1.0–25.0 ml, allowing up to 250.0 ml lysate per line. The maximum load of protein per line is approximately 100 mg and is limited mainly by the capacity of the affinity resin used for capture. Elution fractions of purified samples are collected in 24 deep-well plates on a deck which can hold up to 20 plates. Samples are aspirated from the deck into each syringe valve using the sample-load manifold and then passed through a second set of tubing connecting the purification columns fitted in the primary manifold (Fig. 1 ▶). Using customized software for programming, one operator can input a minimum amount of information for routine parallel purifications. For instance, one can enter the desired elution-buffer concentrations, allowing the software to calculate the appropriate ratios of buffers to mix in each syringe prior to column loading. Tubing in the Protein Maker is made of chemically resistant polyether ether ketone (PEEK) and fluorinated ethylene propylene (FEP) polymers, allowing a wide range of pH buffers and organic solvents to be moved through the lines without damaging the instrument.

In comparison to single-line gravity-flow, HPLC or FPLC systems, the Protein Maker greatly increases output owing to its parallel plumbing and purification capability. Its speed, load capacity and collection volumes make it ideal for reverse-phase, ion-exchange and affinity-based chromatographic separations. In a typical run for SSGCID proteins purified at Emerald BioStructures, immobilized metal-affinity chromatography resin is washed and conditioned for each channel used in the experiment. Protein lysates are typically loaded in volumes of up to 250 ml, washed and eluted prior to analysis by gel or capillary electrophoresis followed by fraction pooling for direct use or subsequent purification steps. Wash buffer containing a minimal amount of imidazole (usually 10 m*M*) assists in removing nonspecifically bound protein from nickel columns and tends to result in higher purity elutions than wash buffer with no imidazole. For cleaved proteins, the 6×His tag and the 6×His-tagged protease used to cleave it are typically removed by one subsequent round of purification on the Protein Maker, collecting the target protein in the initial flowthrough.

Adapting the standard SSGCID purification protocol for the Protein Maker (see §[Sec sec2]2), a single person can purify as many as 48 proteins in an 8 h shift. Using the same protocol in the same amount of time, a lone operator can purify at best four proteins with a single-line FPLC system. For structural genomics work, this level of output is essential for maintaining the high rate of target delivery to crystallization trials and NMR studies. Proteins purified on the Protein Maker for the SSGCID which have resulted in PDB structure depositions include prokaryotic targets from *Bartonella* (PDB entry 3grp; J. Abendroth, T. E. Edwards, B. Sankaran, T. Arakaki & B. L. Staker, unpublished work), *Brucella* (PDB entries 2l3v, 3fq3, 3grk, 3ix6, 3jst, 3oce and 3ocf; R. Barnwal & G. Varani, unpublished work; J. Abendroth, T. E. Edwards, B. Sankaran, T. Arakaki & B. L. Staker, unpublished work; T. E. Edwards, J. Abendroth, B. Sankaran, A. S. Gardberg, T. L. Arakaki & B. L. Staker, unpublished work), *Burkholderia* (PDB entries 3ecd and 3i4e; J. Abendroth, T. E. Edwards, B. Sankaran, T. Arakaki & B. L. Staker, unpublished work; T. E. Edwards, J. Abendroth, T. L. Arakaki & B. Staker, unpublished work), *Mycobacterium* (PDB entries 3gwc and 3hzg; J. Abendroth, T. E. Edwards, B. Sankaran, T. Arakaki & B. L. Staker, unpublished work) and *Rickettsia* (PDB entries 3d53 and 3emj; T. E. Edwards, J. Abendroth, T. L. Arakaki & B. Staker, unpublished work), and eukaryotic proteins from *Encephalitozoon* (PDB entry 3kgb; J. Abendroth, T. E. Edwards, B. Sankaran, T. Arakaki & B. L. Staker, unpublished work), *Babesia* (PDB entries 3i3r, 3k2h, 3kjr and 3nrr; Li *et al.*, 2010 ▶; Begley *et al.*, 2011 ▶) and *Entamoeba* (PDB entries 3lqw, 3sia, 3sib and 3sjs; T. E. Edwards, J. Abendroth, B. Sankaran, A. S. Gardberg, T. L. Arakaki & B. L. Staker, unpublished work; A. S. Gardberg, T. E. Edwards & B. L. Staker, unpublished work), as well as viral proteins from rabies (PDB entry 3oa1; T. E. Edwards, J. Abendroth, B. Sankaran, A. S. Gardberg, T. L. Arakaki & B. L. Staker, unpublished work) and several subtypes of influenza A virus, as described here and elsewhere (Yamada *et al.*, 2010 ▶).

### 3.2. Parallel purification of viral proteins with the Protein Maker

Pandemic outbreaks of influenza have caused millions of deaths throughout history and remain very real threats, as active subtypes currently residing in birds and in swine and other mammalian species possess the potential to infect humans around the globe (Christman *et al.*, 2011 ▶; Sencer, 2011 ▶; Taubenberger & Morens, 2006 ▶; Neumann *et al.*, 2009 ▶). Point mutations in the polymerase basic protein 2 (PB2) of the heterotrimeric viral polymerase and their structural consequences have been linked to host-species specificity and virulence factors in humans (Guilligay *et al.*, 2008 ▶; Mehle & Doudna, 2009 ▶; Subbarao *et al.*, 1993 ▶). This link between PB2 point mutations and transmissibility has prompted further studies of proteins from different subtypes of influenza, including the nomination and approval of various PB2 constructs for study by the SSGCID. Among a wider set of targets, we have characterized the C-terminal PB2 domain (CPB2) from the H1N1 and H5N1 influenza A virus subtypes (Yamada *et al.*, 2010 ▶). H5N1 originated in birds and is responsible for most avian influenza-based fatalities in humans (Li *et al.*, 2004 ▶), while H1N1 caused the outbreak of swine flu in 2009 and is closely related to the 1918 pandemic Spanish flu virus (Taubenberger & Morens, 2006 ▶; Neumann *et al.*, 2009 ▶). Initial success with H5N1 and H1N1 CPB2 proteins informed the design and cloning of genes arising from two other influenza subtypes: H3N2, which was responsible for the 1968 and 1969 Hong Kong flu epidemics, and H7N7, a subtype with wide zoonotic transmissibility and potential for high pathogenicity (Coleman *et al.*, 1968 ▶; Jackson *et al.*, 2010 ▶; Kemink *et al.*, 2004 ▶; Fouchier *et al.*, 2004 ▶). H3N2 is already an increasingly dominant subtype in the annual flu season in North America and is also endemic among livestock pigs in southern China (Gramer *et al.*, 2007 ▶; Richt *et al.*, 2003 ▶). Since pigs can be co-infected with multiple zoonotic influenza A virus subtypes, H3N2 in swine has the potential to emerge in a more virulent form with transmissibility into human populations through genetic reassortment.

In one run of the Protein Maker, five channels were used to purify five different CPB2 constructs (Table 1 ▶) in parallel with other pipeline proteins using our standard SSGCID protocol (see §[Sec sec2]2). In this mode, buffer solutions were aspirated from large common reservoirs into each individual syringe valve, while samples were loaded from 24 deep-well plates using the sample-intake manifold (Fig. 1 ▶). All five constructs were successfully purified from cell lysates as described above (see §[Sec sec2]2), generating highly pure protein (Fig. 2 ▶) for crystallization testing using sparse-matrix screens. Three of the five purified CPB2 proteins yielded crystals (not shown) and a 1.3 Å resolution structure was generated from one of them by X-ray diffraction. This structure was determined by molecular replacement, refined using standard SSGCID protocols (as reported elsewhere in this volume) and deposited in the Protein Data Bank under accession code 3r2v (T. E. Edwards, A. S. Gardberg, B. Sankaran & B. L. Staker, un­published work), for which this communication serves as the first report in the literature. Although previous purifications of CPB2 from H5N1 and H1N1 yielded crystals for 204-residue and 222-residue variants, the only protein which provided well diffracting crystals in this effort was a construct consisting of 216 amino acids (Table 1 ▶). Despite the high sequence identity (>95%) across all constructs, including three previously crystallized CPB2 proteins (Yamada *et al.*, 2010 ▶), only one (InvaB.07055.c.D17) of the five tested led to rapid growth of crystals that diffracted to sufficiently high resolution for structure solution and refinement. Thus, we eliminated the 80% chance of failure (or the fivefold increase in bench time) associated with serial processing of five targets for one successful outcome *via* parallel purification on the Protein Maker.

### 3.3. Scouting lysis-buffer conditions with the Protein Maker

In addition to being a vital asset for high-throughput purification, the Protein Maker can be an invaluable tool for rapid testing of cell-lysis conditions for a wide variety of protein targets. The majority of SSGCID proteins are initially lysed by sonication, followed by nickel-affinity chromatography using genetically engineered histidine tags to bind the protein to the resin (see §[Sec sec2]2). Some targets are insufficiently soluble in the standard cell-lysis buffers for further processing despite high levels of expression. To keep these targets moving through the pipeline, it is often necessary to conduct optimization tests for purification in a rapid parallel fashion. An example of this is cytochrome P450 51 A1 (CYP51A1) from *C. immitis*, a pathogenic fungus that is endemic to North America (Crum *et al.*, 2004 ▶; Galgiani, 1999 ▶; Hector & Laniado-Laborin, 2005 ▶). Inhibition of CYP51A1 with a variety of azole compounds causes accumulation of methylated sterol precursors in fungi, leading to an imbalance in cell-wall stability and reduced fungal growth (Kale & Johnson, 2005 ▶; Sheehan *et al.*, 1999 ▶). Although human CYP51A1 has been structurally characterized (Strushkevich *et al.*, 2010 ▶), together with those of *Mycobacterium tuberculosis* (Podust *et al.*, 2004 ▶) and several protozoan parasites, no CYP51A1 structures from fungal species are available in the PDB. Owing to its potential for structure-guided development of novel antifungals, and as a community-request target, CYP51A1 from *C. immitis* (target ID CoimA.07054.l) was deemed to be a high-value target.

Initial attempts with CYP51A1 showed excellent recombinant protein expression in bacteria, but nearly all of the CYP51A1 remained insoluble using our standard SSGCID cell-lysis methods. We therefore initiated scouting experiments to see if altering the cell-lysis buffer components might provide higher yields from crude lysate. After conducting individual small-scale rapid cell lysis on 12 samples from the same batch of protein, each sample was loaded onto the Protein Maker using a 24 deep-well plate and the sample-intake manifold (Fig. 1 ▶). Keeping the purification buffers identical across all samples allowed a comparative analysis of the different lysis-buffer components used to lyse the cells. SDS–PAGE analysis shows little to no protein in the soluble fraction after lysis, suggesting that CYP51A1 is only soluble under certain conditions. The most promising results were obtained with lysis buffer containing 3-[(3-cholamidopropyl)­dimethylammonio]-1-propanesulfonate (CHAPS) or *n*-­octyl-β-d-glucopyranoside (BOG) with 500 m*M* salt at the lowest pH tested (Fig. 3 ▶). CHAPS and BOG are nonionic detergents that are favored in membrane-protein extraction, partly owing to the relative ease of their removal further downstream in the purification. BOG is especially favored when working with membrane proteins because of its ability to soften the phospholipid layer (Markovic Housley & Garavito, 1986 ▶; Garavito & Ferguson-Miller, 2001 ▶). In this case, the detergents are likely to have contributed to the breakdown of cell membranes, releasing greater amounts of recombinant protein into solution (Fig. 3 ▶). With a putative transmembrane helix N-terminal to the canonical P450 domain of our construct, it is also possible that detergents are necessary to help solubilize the target protein. Thus, results from this test have provided optimal cell-lysis buffer conditions for further work on scale-up and purification of CYP51A1 and have led to further work in gene optimization.

## 4. Conclusions

As more genomes are sequenced, the range of uncharacterized biological targets of interest expands, increasing the need to rapidly clone, express and purify targets for structural and functional genomics. With more than 5000 targets now selected for investigation by the SSGCID, it has become essential to utilize automated laboratory systems capable of producing large quantities of pure crystallization-grade protein. A single person using a single-channel FPLC system in our facility can fully process up to four protein targets in a week from lysis to size-exclusion chromatography following standard SSGCID practices and protocols. The same individual can process up to 40 proteins per week with the Protein Maker using the same protocols for individual sample lysis and staggered-loop consecutive injections for single-line size-exclusion chromatography. This tenfold increase in sampling capacity allows the processing of many more constructs per target, thereby improving the chance that a protein of interest becomes structurally characterized. Without the Protein Maker, serial purification of five unique CBP2 constructs would be likely to have taken two weeks of effort, fully occupying the instrument and a single researcher for that duration. In such a scenario, construct design may have been limited to residue lengths which previously worked for CBP2 from H1N1 and H5N1, constructs which to date have not generated crystals for H3N2. Although only two conditions appeared to increase the yield of our CYP51A1 protein target, this information was sufficient to improve lysis conditions for milligram-scale purification. The identification of key components in the cell-lysis buffer also prompted a more thorough investigation of the construct and has led to second-generation deletion mutants to obtain the P450 domain of this important antifungal drug target. Whether for method development or high-throughput processing, the parallel purification options afforded through the Protein Maker has made it a valuable asset for the SSGCID pipeline and the determination of structures for infectious disease research.

## Figures and Tables

**Table 1:** Constructs of the C-terminal domain of polymerase basic protein 2 (PB2) derived from different subtypes of influenza virus

Target database ID	Construct	Residues	Source	Subtype	Results
InvaB.07055.c	D16	538–741	Yokohama 2017 (2003)	H3N2	Crystals
InvaB.07055.c	D17	538–753	Yokohama 2017 (2003)	H3N2	PDB entry 3r2v
InvaC.07055.b	D15	538–759	Prague 1 (1956)	H7N7	Crystals
InvaC.07055.b	D16	538–741	Prague 1 (1956)	H7N7	Purified
InvaC.07055.b	D17	538–753	Prague 1 (1956)	H7N7	Purified
InvaA.07055.a†	D16	538–741	Vietnam 1203 (2004)	H5N1	PDB entry 3kc6
InvaA.07055.a†	D15	538–759	Vietnam 1203 (2004)	H5N1	PDB entry 3l56
InvaE.07055.a†	D16	538–741	Mexico INDRE4487 (2009)	H1N1	PDB entry 3khw

† Results previously reported elsewhere (Yamada *et al.*, 2010 ▶).

**Table 2:** Condition grid for cell-lysis buffer testing of recombinantly expressed fungal cytochrome P450 (CYP51A1 from *C. immitis*; CoimA.07054.l)

	A	B	C	D
1	50 m*M* MES, 250 m*M* NaCl, 5% glycerol, 0.5 m*M* TCEP **pH 6.0**	50 m*M* MES, 1 *M* NaCl, 5% glycerol, 0.5 m*M* TCEP **pH 6.0**	50 m*M* MES, 500 m*M* NaCl, 5% glycerol, 0.5 m*M* TCEP, 1% CHAPS **pH 6.0**	50 m*M* MES, 500 m*M* NaCl, 5% glycerol, 0.5 m*M* TCEP, 1% BOG **pH 6.0**
2	50 m*M* HEPES, 250 m*M* NaCl, 5% glycerol, 0.5 m*M* TCEP **pH 7.5**	50 m*M* HEPES, 1 *M* NaCl, 5% glycerol, 0.5 m*M* TCEP **pH 7.5**	50 m*M* HEPES, 500 m*M* NaCl, 5% glycerol, 0.5 m*M* TCEP, 1% CHAPS **pH 7.5**	50 m*M* HEPES, 500 m*M* NaCl, 5% glycerol, 0.5 mM TCEP, 1% BOG **pH 7.5**
3	50 m*M* Tris, 250 m*M* NaCl, 5% glycerol, 0.5 m*M* TCEP **pH 8.0**	50 m*M* Tris, 1 *M* NaCl, 5% glycerol, 0.5 m*M* TCEP **pH 8.0**	50 m*M* Tris, 500 m*M* NaCl, 5% glycerol, 0.5 m*M* TCEP, 1% CHAPS **pH 8.0**	50 m*M* Tris, 500 m*M* NaCl, 5% glycerol, 0.5 m*M* TCEP, 1% BOG **pH 8.0**

## References

[bb3] Begley, D. W., Edwards, T. E., Raymond, A. C., Smith, E. R., Hartley, R. C., Abendroth, J., Sankaran, B., Lorimer, D. D., Myler, P. J., Staker, B. L. & Stewart, L. J. (2011). *Acta Cryst.* F**67**, 1070–1077. 10.1107/S1744309111029009 PMC3169404 21904052

[bb4] Berman, H., Henrick, K. & Nakamura, H. (2003). *Nature Struct. Biol.* **10**, 980. 10.1038/nsb1203-980 14634627

[bb5] Berman, H. M., Westbrook, J., Feng, Z., Gilliland, G., Bhat, T. N., Weissig, H., Shindyalov, I. N. & Bourne, P. E. (2000). *Nucleic Acids Res.* **28**, 235–242. 10.1093/nar/28.1.235 PMC102472 10592235

[bb6] Bonanno, J. B. *et al.* (2005). *J. Struct. Funct. Genomics*, **6**, 225–232. 10.1007/s10969-005-6827-0 16211523

[bb7] Christman, M. C., Kedwaii, A., Xu, J., Donis, R. O. & Lu, G. (2011). *Infect. Genet. Evol.* **11**, 803–811. 10.1016/j.meegid.2011.02.021 PMC3141221 21382522

[bb8] Coleman, M. T., Dowdle, W. R., Pereira, H. G., Schild, G. C. & Chang, W. K. (1968). *Lancet*, **2**, 1384–1386. 10.1016/s0140-6736(68)92683-4 4177941

[bb9] Crum, N. F., Lederman, E. R., Stafford, C. M., Parrish, J. S. & Wallace, M. R. (2004). *Medicine*, **83**, 149–175. 10.1097/01.md.0000126762.91040.fd 15118543

[bb10] Cymborowski, M., Klimecka, M., Chruszcz, M., Zimmerman, M. D., Shumilin, I. A., Borek, D., Lazarski, K., Joachimiak, A., Otwinowski, Z., Anderson, W. & Minor, W. (2010). *J. Struct. Funct. Genomics*, **11**, 211–221. 10.1007/s10969-010-9092-9 PMC2921494 20526815

[bb11] Dieckman, L., Gu, M., Stols, L., Donnelly, M. I. & Collart, F. R. (2002). *Protein Expr. Purif.* **25**, 1–7. 10.1006/prep.2001.1602 12071692

[bb15] Elsliger, M.-A., Deacon, A. M., Godzik, A., Lesley, S. A., Wooley, J., Wüthrich, K. & Wilson, I. A. (2010). *Acta Cryst.* F**66**, 1137–1142. 10.1107/S1744309110038212 PMC2954196 20944202

[bb16] Fouchier, R. A., Schneeberger, P. M., Rozendaal, F. W., Broekman, J. M., Kemink, S. A., Munster, V., Kuiken, T., Rimmelzwaan, G. F., Schutten, M., Van Doornum, G. J., Koch, G., Bosman, A., Koopmans, M. & Osterhaus, A. D. (2004). *Proc. Natl Acad. Sci. USA*, **101**, 1356–1361. 10.1073/pnas.0308352100 PMC337057 14745020

[bb17] Galgiani, J. N. (1999). *Ann. Intern. Med.* **130**, 293–300. 10.7326/0003-4819-130-4-199902160-00015 10068388

[bb18] Garavito, R. M. & Ferguson-Miller, S. (2001). *J. Biol. Chem.* **276**, 32403–32406. 10.1074/jbc.R100031200 11432878

[bb20] Gramer, M. R., Lee, J. H., Choi, Y. K., Goyal, S. M. & Joo, H. S. (2007). *Can. J. Vet. Res.* **71**, 201–206. PMC1899866 17695595

[bb21] Guilligay, D., Tarendeau, F., Resa-Infante, P., Coloma, R., Crepin, T., Sehr, P., Lewis, J., Ruigrok, R. W., Ortin, J., Hart, D. J. & Cusack, S. (2008). *Nature Struct. Mol. Biol.* **15**, 500–506. 10.1038/nsmb.1421 18454157

[bb22] Hector, R. F. & Laniado-Laborin, R. (2005). *PLoS Med.* **2**, e2. 10.1371/journal.pmed.0020002 PMC545195 15696207

[bb23] Jackson, C., Vynnycky, E. & Mangtani, P. (2010). *Am. J. Epidemiol.* **171**, 465–478. 10.1093/aje/kwp394 PMC2816729 20007674

[bb24] Kale, P. & Johnson, L. B. (2005). *Drugs Today*, **41**, 91–105. 10.1358/dot.2005.41.2.882661 15821782

[bb25] Kemink, S. A., Fouchier, R. A., Rozendaal, F. W., Broekman, J. M., Koopmans, M., Osterhaus, A. D. & Schneeberger, P. M. (2004). *Ned. Tijdschr. Geneeskd.* **148**, 2190–2194. 15559415

[bb26] Kim, Y. *et al.* (2008). *Adv. Protein Chem. Struct. Biol.* **75**, 85–105. 10.1016/S0065-3233(07)75003-9 PMC3366499 20731990

[bb27] Li, L., Du, W. & Ismagilov, R. F. (2010). *J. Am. Chem. Soc.* **132**, 112–119. 10.1021/ja908558m PMC2805062 20000709

[bb28] Li, K. S. *et al.* (2004). *Nature (London)*, **430**, 209–213.

[bb29] Lorimer, D., Raymond, A., Walchli, J., Mixon, M., Barrow, A., Wallace, E., Grice, R., Burgin, A. & Stewart, L. (2009). *BMC Biotechnol.* **9**, 36. 10.1186/1472-6750-9-36 PMC2681465 19383142

[bb30] Markovic Housley, Z. & Garavito, R. M. (1986). *Biochim. Biophys. Acta*, **869**, 158–170. 10.1016/0167-4838(86)90290-6 3002479

[bb31] Mehle, A. & Doudna, J. A. (2009). *Proc. Natl Acad. Sci. USA*, **106**, 21312–21316. 10.1073/pnas.0911915106 PMC2789757 19995968

[bb49] Mossessova, E. & Lima, C. D. (2000). *Mol. Cell*, **5**, 865–876. 10.1016/s1097-2765(00)80326-3 10882122

[bb32] Myler, P. J., Stacy, R., Stewart, L., Staker, B. L., Van Voorhis, W. C., Varani, G. & Buchko, G. W. (2009). *Infect. Disord. Drug Targets*, **9**, 493–506. 10.2174/187152609789105687 PMC2857597 19594426

[bb33] Neumann, G., Noda, T. & Kawaoka, Y. (2009). *Nature (London)*, **459**, 931–939. 10.1038/nature08157 PMC2873852 19525932

[bb34] Podust, L. M., Yermalitskaya, L. V., Lepesheva, G. I., Podust, V. N., Dalmasso, E. A. & Waterman, M. R. (2004). *Structure*, **12**, 1937–1945. 10.1016/j.str.2004.08.009 15530358

[bb35] Richt, J. A., Lager, K. M., Janke, B. H., Woods, R. D., Webster, R. G. & Webby, R. J. (2003). *J. Clin. Microbiol.* **41**, 3198–3205. 10.1128/JCM.41.7.3198-3205.2003 PMC165376 12843064

[bb36] Sencer, D. J. (2011). *Clin. Infect. Dis.* **52**, S4–S7. 10.1093/cid/ciq006 21342898

[bb37] Sheehan, D. J., Hitchcock, C. A. & Sibley, C. M. (1999). *Clin. Microbiol. Rev.* **12**, 40–79. 10.1128/cmr.12.1.40 PMC88906 9880474

[bb38] Steen, J., Uhlén, M., Hober, S. & Ottosson, J. (2006). *Protein Expr. Purif.* **46**, 173–178. 10.1016/j.pep.2005.12.010 16483795

[bb39] Stols, L., Gu, M., Dieckman, L., Raffen, R., Collart, F. R. & Donnelly, M. I. (2002). *Protein Expr. Purif.* **25**, 8–15. 10.1006/prep.2001.1603 12071693

[bb40] Strushkevich, N., Usanov, S. A. & Park, H. W. (2010). *J. Mol. Biol.* **397**, 1067–1078. 10.1016/j.jmb.2010.01.075 20149798

[bb41] Subbarao, E. K., London, W. & Murphy, B. R. (1993). *J. Virol.* **67**, 1761–1764. 10.1128/jvi.67.4.1761-1764.1993 PMC240216 8445709

[bb42] Tarendeau, F., Crepin, T., Guilligay, D., Ruigrok, R. W., Cusack, S. & Hart, D. J. (2008). *PLoS Pathog.* **4**, e1000136. 10.1371/journal.ppat.1000136 PMC2515345 18769709

[bb43] Taubenberger, J. K. & Morens, D. M. (2006). *Emerg. Infect. Dis.* **12**, 15–22. 10.3201/eid1201.050979 PMC3291398 16494711

[bb44] Van Voorhis, W. C., Hol, W. G., Myler, P. J. & Stewart, L. J. (2009). *PLoS Comput. Biol.* **5**, e1000530. 10.1371/journal.pcbi.1000530 PMC2756625 19855826

[bb45] Walls, D., Loughran, S. T., Madadlou, A., O’Sullivan, S. & Sheehan, D. (2011). *Protein Chromatography*, pp. 439–447. Totowa: Humana Press.

[bb46] Watson, J. D., Sanderson, S., Ezersky, A., Savchenko, A., Edwards, A., Orengo, C., Joachimiak, A., Laskowski, R. A. & Thornton, J. M. (2007). *J. Mol. Biol.* **367**, 1511–1522. 10.1016/j.jmb.2007.01.063 PMC2566530 17316683

[bb47] Xiao, R. *et al.* (2010). *J. Struct. Biol.* **172**, 21–33. 10.1016/j.jsb.2010.07.011 PMC4110633 20688167

[bb48] Yamada, S. *et al.* (2010). *PLoS Pathog.* **6**, e1001034. 10.1371/journal.ppat.1001034 PMC2916879 20700447

